# Purification and biocompatibility of fermented hyaluronic acid for its applications to biomaterials

**DOI:** 10.1186/2055-7124-18-6

**Published:** 2014-06-13

**Authors:** Sungchul Choi, Woncheol Choi, Sekweon Kim, Su-Yeon Lee, Insup Noh, Chan-Wha Kim

**Affiliations:** School of Life Science and Biotechnology, Korea University, Seoul, 136-701 Republic of Korea; Department of Bioplant, Hanmi Pharm. Co, Pyeongtaek, 451-805 Korea; Department of Chemical Engineering, Seoul National University of Science and Technology, Seoul, 139-743 Korea; Convergence Institute of Biomedical Engineering and Biomaterials, Seoul National University of Science and Technology, Seoul, 139-743 Korea

**Keywords:** Hyaluronic acid, Purification, Activated carbon, Adsorption, Biomaterials

## Abstract

**Background:**

Hyaluronic acid (HA) is of importance due to its diverse applications in pharmaceuticals and medical devices such as dermal filler, adhesion barriers, carrier for cells and bioactive molecules as well as scaffold biomaterials for tissue engineering. Evaluations of purification and biocompatibility of HA are required for its applications to biomaterials.

**Results:**

After synthesizing HA by fermentation of *streptococcus zooepidemicus* for 25 hr, extensively purification of the fermented broth was performed to remove impurities using a filtration process for insoluble components and cells, and diverse adsorbents for soluble impurities. Its *in vitro* biocompatibility has been evaluated by measurement of cell counting and assay of cell live and dead. 60% yield of white HA powder was obtained, having 15–17 dL/g intrinsic viscosity with a molecular weight of approximately 1,000 kDa. While low molecular weight impurities and insoluble impurities were successfully removed using a ultrafiltration membrane with 50 KDa molecular weight cut, endotoxins, high molecular weight proteins and nucleic acids were removed from the broth by employing adsorbents such as alumina and activated carbons. Alumina showed the best results for the removal of endotoxins, all of the activated carbons were very effective in the removal of high molecular weight proteins and nucleic acids. The purified HA solution showed excellent cell compatibility with no cell damages as observed by both measurement of cell proliferation and observation of cell viability.

**Conclusions:**

We obtained high molecular weight HA with excellent biocompatibility as judged by both measurement of cell proliferation and viability, indicating high possibility of its applications to biomaterials.

## Background

Hyaluronic acid (HA) is a linear anionic, non-sulfated, high molecular weight glycosaminoglycan with alternating D-glucuronic acid and *N*-acetyl-D-glucosamine. HA is distributed natively as a component of extracellular matrix in connective, epithelial and neutral tissues in body. HA has several important physico-chemical and biological properties including lubricity, visco-elasticity, water retention, biocompatibility, cell proliferation, morphogenesis, inflammation and wound repair as well as specific signal transduction and cellular interactions through cell surface receptors such as CD44, CD54 and CD168 [1–3]. HA can be degraded by both reactive oxygen intermediates and hyaluronases, which are synthesized by macrophages, fibroblasts and endothelial cells [4, 5].

HA has been produced by either extraction from rooster combs, the highly viscous vitreous humor of bovine eyes [6, 7] and human umbilical cord or bacterial fermentation of group C streptococci, namely hemolytic streptococci [8]. Purification of medium containing HA products has been achieved using a variety of different methods [9–14], including filtration and adsorption. These methods normally result in the purification of HA with molecular weights ranging from 10^4^ to 10^7^ Da [14]. Various forms of purified HA have been used in different commercial products such as cosmetics [15], eye drops [16], food additives [17], medical devices [1], pharmaceutics [18–20], tissue engineering [21, 22] and cell therapy [23]. For example, Viscoat has been used as a viscosurgical agent by Alcon Co. (TX, USA) and for visco-supplementation in arthritic joints by Seikagaku Co. (Tokyo, Japan). In addition, Fidia in Italy utilized HA with moderate molecular size as a visco-supplement, Genzyme Co. (MA, USA) and Q-Meds used cross-linked HA products as visco-supplements. HA is currently being investigated for use as a bioactive material for plastic fillers to eliminate facial wrinkles, a carrier to deliver stem cells, bioactive materials to treat specific diseases and a scaffold for tissue engineering of bone, cartilage, blood vessel and nerves [21, 22].

**Figure 1:**
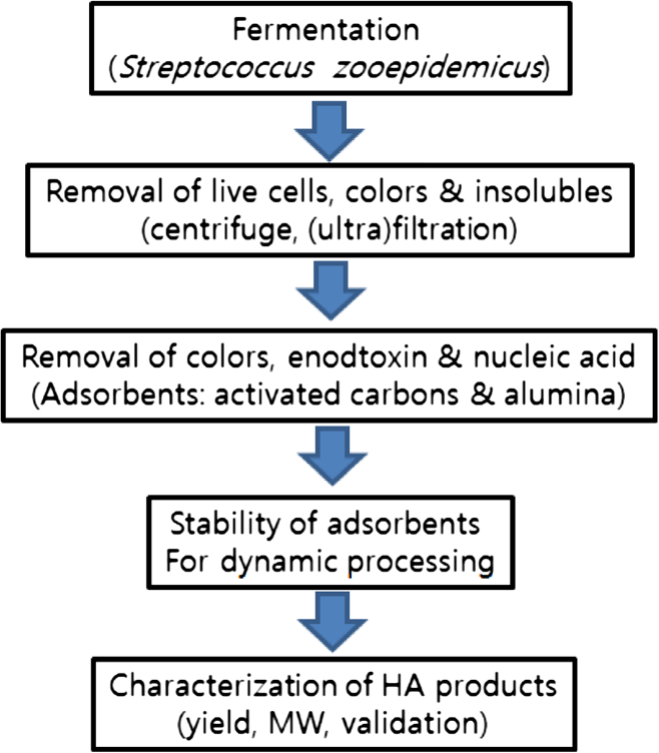
**Schematic processes of bacterial fermentation and HA separations and characterizations.**

Recently demands for HA products from bacterial fermentation have significantly increased because of both their increased uses as medical devices and the immune issues that occurred from the use of animal based HA. Due to the both high price of HA and the high standard requirements of its applications in medical products, high quality HA products rather than high quantity have been the primary criteria used when selecting the bacterial strains used for HA production and the methods of HA purification. In this study, we examined the effects of various adsorbents such as activated carbons and alumina on the purification of fermented HA broth as well as biocompatibility tests. Schematic processes of bacterial fermentation and HA separations and characterizations are shown in Figure 1. The HA obtained in this study may expand the potential application of HA in biomaterials targeting on the areas of cell therapy, tissue engineering and medical devices.

## Methods

### Materials

*Streptococcus zooepidemicus*, mutant bacterial strains, were employed in the fermentation experiments. The yeast extract and sodium chloride were purchased from Beckton Dickinson (USA) and Merk Co. (Germany), respectively, and glucose and potassium phosphate were obtained from Sigma Co. (USA). L-Glutamate and membrane filter were obtained from Daesang Co. (Korea) and Begerow (Germany), respectively. Ultra-filtration membrane filters and diafiltration membranes with a molecular weight cut offs 30 and 50 KDa were purchased from Milipore Co (MA, USA). The different adsorbents including alumina and eight activated carbons were purchased from Baikowski Co. (Japan) and Norit Co. (Netherland), respectively. The specific physicochemical properties such as BET, pH, moisture, particle size, iodine number and morphologies of the absorbents are described in Table 1.

**Table 1:** **Physical properties of the adsorbents employed in the adsorption process**

Model name	Morphology	Particle size (μm; d10, d50, d90)*	Iodine number	Methylene blue adsorption (g/100 g)	B.E.T.** (m ^2^/g)	pH	Moisture (%)
Darco KB-B	Powder	4, 15, 40	-	42	2050	2	≤15%
Norit CN1	Powder	7, 30, 76	-	29	1400	5.5 ~ 8	≤15%
Norit SX Plus	Powder	6, 20, 80	1050	22	1100	Neutral	≤10%
Norit SX 1G	Powder	5, 25, 90	900	18	1000	Neutral	≤10%
Norit C Gran	Granule	0.85 mm ~ 1.7 mm***	-	30	1400	2 ~ 8	≤15%
Norit GAC 1240^+^	Granule	0.43 mm ~ 2.0 mm***	1025	22	1125	Neutral	≤3%
Norit C Extra USP	Powder	4, 23, 100	1100	22	1200	Neutral	≤10%
Norit A Supra EUR	Powder	4, 20, 100	1550	41	1700	Neutral	≤10%
Alumina	Powder	2.5, 6, 10	-	-	103	-	-

*The particle sizes (diameter) indicated that their actual sizes are less than those given.

**Total surface area.

***More than 95% have the granule sizes indicated.

### Fermentation and analysis of the properties of the fermenting broth

*Streptococcus zooepidemicus* was fermented in a bioreactor (5 L, Marubishi Inc.) at 37°C and 300 rpm for 25 hr with 1.0 vvm aeration to obtain hyaluronic acid, which have been reported well by numerous companies and researchers, including Akdamar HA and Volpi N [11, 12]. The complex media under an aerobic condition that was used as the fermenting broth consisted of yeast extract (15 ~ 25 g/L), potassium phosphate (2.5 g/L), glucose (60 ~ 80 g/L), magnesium sulfate (3 g/L), sodium chloride (5 g/L) and L-glutamate (7 g/L). The residual L-glucose in the fermenting broth was analyzed using 1 ml in a biochemical analyzer (MBI 7200 model, YSI Co., USA) and the optical density of the broth was measured with a Spectrophotometer (UV2450, Simadzu Co., Japan) at an wavelength of 600 nm after a 10 fold dilution. The broth’s viscosity was measured using a rotary viscometer (LV type, Brookfield Co.) and 400 mL of the solution.

### Purification

#### Removal of cells

The bacterial strains and insoluble impurities in the highly viscous broth were removed by filtering with a depth filter (KD7, Begerow Co.) that had a 1.5 μm nominal retention rate after mixing with either 1, 3 or 5% diatomaceous earth (v/v). The efficacy of the broth filtration was confirmed by measuring the presence of bacteria with a Spectrophotometer (UV2450, Simadzu Co., Japan) at a wavelength of 600 nm.

#### Diafiltration purification

Diafiltration was used for the removal of broth components, metabolic products and low molecular weight impurities from 5 L of broth. This process was conducted using a tangential flow filtration method by employing an Ultra Filter (Pellicon2, Millipore, USA) after removal of the bacterial strains and insoluble impurities. The sample was then diluted by a factor of 2 using deionized water. Diafiltration was performed by pumping the solution through the poly(ether sulphone) ultrafilter cassette with a dimension of 0.5 m^2^ and a molecular weight cut-off of either 30 or 50 kDa (PR sterile 40, Begerow Inc., Germany). After defining the number of the diafiltration purification as the number of alternating processes of both concentration and dilution, both the permeate and conductivity and the concentration of residual lactate were measured using a conductivity meter (Seveneasy; Mettler-Toledo, Swizerland) and the biochemical analyzer (MBI 7200; YSI Inc., USA), respectively. The concentration of residual lactates was measured by feeding 1 mL into the biochemical analyzer and measuring the electrical signals from the residual lactates, which were produced as a metabolic byproduct during the fermenting process. The electrical signals were measured by the biochemical analyzer during induction of lactates degradation by L-lactate oxidases. The volume of the permeate solution filtered from the membrane was measured in a dimension of L/h/m^2^ to check the buffer change time during diafiltration.

#### Purification by adsorbents

High molecular weight impurities were removed by adding different adsorbents to the diafiltrated broth. After addition of 2% adsorbent (w/v) into 1,000 mL of the diafiltrated broth, the solution was stirred with a stirrer (RZR2021, Heidolph Inc., Germany) for 10 hr. The adsorbents were then removed using a 0.45 μm filter (PR sterile 40, Begerow Inc., Germany).

#### Recovery of the hyaluronic acid in powder

Solidification of the HA sample was achieved by adding 3 L of acetone to the adsorption-processed solution. The HA powder was recovered and dried after filtration of the solidified HA with a 100 μm mesh (CISA, Spain). The HA yield was determined to be about 60%, which was measured by comparing the weight of the dry HA with that of the fermented weight.

### Analysis

Verification of hyaluronic acid powder was performed using the analytical methods described in both the European pharmacopoeia and Korean pharmacopoeia, which included measuring the residual levels of endotoxins, proteins, nucleic acids and small particles.

#### Endotoxin analysis

The kinetic chromogenic method (Kinetic-QCL Chromogenic LAL assay, Lonza, USA) was used to measure the amount of residual endotoxin. After syntheses of both standard and test solutions according to the procedures suggested by the vendor, the LAL kinetic chromogenic reagent was added and its optical density was measured at a wavelength of 405 nm using a microplate meter. Two mL standard solutions were obtained by diluting the standards to 0.005, 0.05, 0.5, 5 EU/mL with the LAL reagent water (Lonza Co., USA). The test solution was prepared by diluting the sample solution to the lowest possible level to minimize the effect of inhibition factors. The LAL reagent was prepared by adding up LAL reagent water to the LAL kinetic chromogenic reagent.

#### Protein analysis

Examination of residual high molecular weight proteins in the hyaluronic acid powder was performed according to the protein tests recommended by the Korean pharmacopoeia. The test solution was prepared by dissolving 0.1 g HA powder in 20 mL deionized water. BSA was used as the reference solution and the BSA concentrations used were 1, 3, 5, 7 and 10%. Test and standard solutions were reacted with 2.5 mL cupri-tartaric solution for 10 min. After reacting with 0.5 mL of the phosphomolybdotunstic reagent for 30 min, the solution’s optical density was measured at a wavelength of 750 nm, which was used to calculate the concentrations of residual proteins. The phosphomolybdotunstic reagent was obtained by refluxing for 10 hr in a reflux condenser after the addition of 100 g sodium tunstate and 25 g sodium molybdate, and then 100 mL hydrogen chloride and 50 mL phosphonic acid. To remove excess bromine, the sample was boiled for 15 min after the addition of 150 g lithum sulfate, 50 mL water and bromine. Water was added to a final volume of 1,000 mL and then the sample was filtered. The cupri-tartaric solution was prepared by mixing 50 mL sodium carbonate with 0.1 mL of a solution that was previously prepared by dissolving 1.0 g copper sulfate and 2.0 g sodium tartarate in 100 mL deionized water. The 100 mL sodium carbonate solution was obtained by dissolving 4.0 g sodium carbonate in 0.2 M NaOH.

#### Nucleic acid analysis

Residual nucleic acid was analyzed in either a liquid or powder state. The amount of nucleic acid in the processing broth was obtained by measuring the optical density of a 1 mL solution at a wavelength of 260 nm using the Spectrophotometer (Simadzu Inc., Japan) and the amount of nucleic acid in the powder was determined by adding the powder to 30 mL 0.9% sodium chloride solution and then measuring absorbance at 260 nm.

#### Measurement of micro-particles in the processing broth

Micro-particles in the processing broth after treatment with diverse activated carbons were measured using a liquid particle counter (LS-200, Particle Measuring Systems Inc., USA). In this analysis, 20 mL test solution either with or without HA was added to the liquid particle counter as well as solution containing 2% of the activated carbons were fed in either granule or powder form.

#### Molecular weight of HA

The molecular weight of the dry HA was measured using a Multi Angle Laser Light Scattering (MALS) detector (DAWN, Wyatt Technology Co., US) and high performance liquid chromatography (HPLC) (1200 series, Agilent, Germany) (column: TSK G6000 PWXL, Tosoh, Japan). A 0.02 ~ 0.03% HA solution was filtered through a 0.45 μm filter (Acrovisk, PALL, USA) and then injected into the HPLC system with a glass syringe. The molecular weights of the HA compounds were measured using a multi-angle light scattering (MALS) detector.

#### Intrinsic viscosity

40 mL 0.005%, 0.010%, 0.015% and 0.020% HA solutions were synthesized by dissolving the dry HA powder in buffer solution (0.01 M) with 0.15 M sodium chloride at pH 7. Relative viscosity was obtained by measuring the flow time of the HA solution with a capillary viscometer (Type 501 01, SI Analytics GmbH, Germany) and linear least-square regression of the Martin equation.

### *In vitro*evaluation of HA samples

#### In vitro cell culture

MC3T3 cells, osteoblast precursor cell line, were *in vitro* cultured in α-MEM media (Sigma Aldrich, USA) containing 10% fetal bovine serum and penicillin-streptomycin (100 IU/mL) and gentamycin (2 L/mL) in an incubator with 5% CO_2_ at 37°C. Sterilized HA solution (Hyalrheuma Inj.; Hanmi Pharmaceutical Co., Pyeongtaek, Korea) was loaded on a 24 well polystyrene tissue culture plate (Nunc Co., USA). MC3T3 cells, 10,000 cells/well, were seeded on the surface of 200 μl HA solution and cultured under static conditions in an incubator with 5% CO_2_ at 37°C for 7 days.

#### Cell proliferation assay

Cell adhesion and proliferation on the HA solution was quantitatively measured by counting the number of cells with a cell counting kit-8 (CCK-8; Dojindo: Japan) by a microplate reader (Tecan: Australia). 100 μL solution of CCK-8 was inserted into the 1 mL cell culture medium and then the cell culture plate was incubated in 5% CO_2_ incubator at 37°C. After 4 hr, 100 μL α-MEM with CCK-8 was aliquoted into a 96 well plate and then the optical its density was measured at the wavelength of 450 nm [23].

#### In vitro cell viability by live & dead assay

For observation of cytotoxicity of the HA solution, cell viability on the HA solution was evaluated by Live & Dead assay after *in vitro* cell culture with MC3T3 cells for 7 days according to our previous report, by staining with fluororescence dye [24].

## Results and discussion

### Bacterial fermentation for HA production

Bacterial fermentation of *streptococcus zooepidemicus* in the L-glucose bioreactor continued until complete consumption of L-glucose in the culture broth. The fermentor was operated at 37°C and 300 rpm for 25 hr with 1.0 vvm aeration, which resulted in the production of 6.0 ~ 6.5 g HA/L. The optical density of the fermented solution ranged from 8.0 to 12.0 and the broth viscosity processed at 35°C and 21 spindles was as approximately 8000 cP. The molecular weight of the HA obtained was determined to be 900 ~ 1,100 kDa.

### HA purification of processing broth

#### Removal of bacteria and insoluble impurities

Bacteria strains and insoluble impurities were removed by filtration of the fermented broth using different retention rates of the fibrillated cellulose fibers filter sheets and the addition of different concentrations of earth soil. The filter had three retention rate sizes of 0.6, 1.0. 1.5 μm, when 1, 3 and 5% earth soil concentrations (w/v) were used. The effects of filtration on HA purification are clearly shown in Table 2. Before filtration, the fermented solution had an optical density of 5.30. However, after filtration, the optical densities were less than 0.1 for all the samples. By increasing the earth oil concentrations, the flow rates increased significantly (2.5, 5.3 and 10.0 L/hr at earth oil concentrations of 1, 3 and 5% earth oils) when filters with a nominal retention rate of 0.6 μm were used. These increases in the flow rates were applied to all other conditions such as 1.0 and 1.5 nominal retention rates of the filter sheets.

**Table 2:** **Removal of strains by filtration conditions**

	Filtration conditions		After filtration	
	Nominal retention rate (μm)	Earth oil conc. (%)	OD (260 nm)	Flow rates (L/hr)
Before filtration	-	-	5.30	-
After filtration	0.6	1	0.06	2.5
	0.6	3	0.05	5.3
	0.6	5	0.03	10
	1.0	1	0.05	3.7
	1.0	3	0.07	6.2
	1.0	5	0.03	9.3
	1.5	1	0.06	5.8
	1.5	3	0.02	8.5
	1.5	5	0.02	13.3

#### HA purification with diafiltration method

Next we removed impurities such as culture products and broth components from the bacteria-free broth through filtration. The extent of impurity removal was evaluated by measuring membrane conductivity and flow rates over the membrane pores, and the number of diafiltration cycles.

##### Measurement of conductivity and flow rates of broth

**Figure 2:**
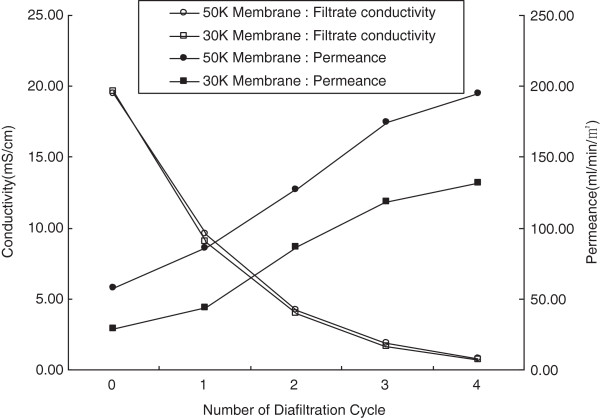
**Conductivities and permeance of filtrate through the membrane filters over different cycles of diafiltration.**

We employed poly(ether sulphone) membranes with two kinds of pore sizes, i.e. with molecular weight cut-off sizes of both 30 and 50 KD, to measure the conductivity and permeance of the membrane filters (Figure 2). The conductivities of the two membranes were similar to each other at the same number of diafiltration cycles, but the values significantly decreased as the number of diafiltration cycles was increased. More specifically, the conductivities of the membranes with the 30 KD molecular weight cut-off size decreased from 19.6 to 9.01, 4.01, 1.7 and 0.7 ms/cm for diafiltration cycles of 1, 2, 3 and 4, respectively. However, the permeance of the membrane filters were displayed a significant different behavior than the conductivities. As expected, the peremances of the membrane filters for both molecular weight cut-off sizes, i.e. 30 and 50 KDa, increased when the number of diafiltration cycles was increased. The permeance of the membrane filters with a 50 KDa molecular weight cut-off size increased from 3.5 to 5.1, 7.6, 10.5 and 11.7 L/hr/m^2^, while the permeance of the 30 kDa molecular weight cut-off size increased from 1.7 to 2.6, 5.2, 7.1 and 7.9 L/hr/m^2^. These results indicated that the membrane filters with higher molecular weight cut-off sizes and samples subject to more diafiltration cycles had higher rates of permeate flow. The reason for the increase in permeances by repeated diafiltrations was previously shown to be due to the removal of air entrapped in the membrane pores [24].

##### Removal of impurities by diafiltrations

**Figure 3:**
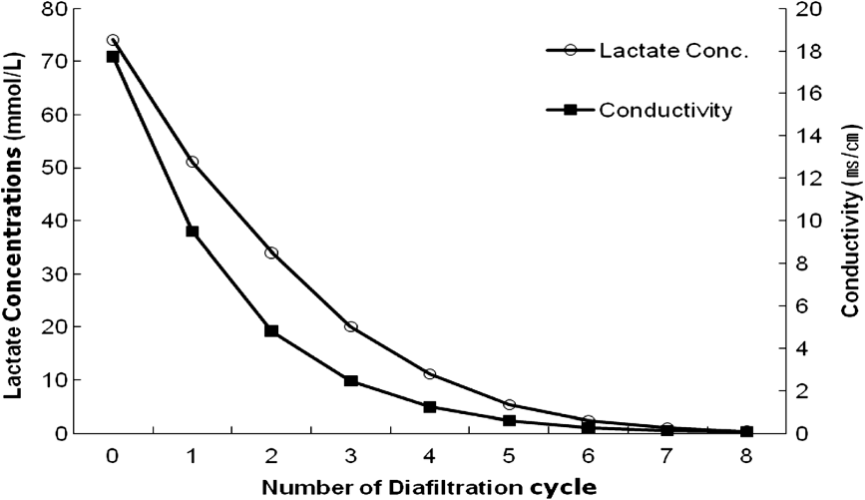
**Membrane conductivity and lactate concentrations in the permeates after diafiltrations.**

 Next we measured membrane conductivity and lactate concentrations in the broth samples that had been subjected to a different number of diafiltration cycles (Figure 3). As expected, both the conductivity of the membrane filter and the concentrations of the sample decreased significantly when the number of diafiltration cycles was increased. When the conductivities decreased from 17.7 before diafiltration to 9.5, 4.8, 2.5, 1.2, 0.6, 0.3 and 0.1 by increasing the filtration cycles from 1 to 8, the concentrations of lactates in the samples decreased from 74.1 to 51.0, 34.0, 20.1, 11.0, 5.4, 2.3, 1.0 and 0.4, respectively. These results showed that lactate impurities decreased to approximately 1% by repeating diafiltration 7 times.

#### Purification

The medium solutions that had been diafiltrated still contained significant amounts of impurities including colors (67.44 EU/mL) and endotoxins (77.73 μg/mL). Therefore, the filtrated broth was further purified using various adsorbents as described below.

##### Removal of endotoxin

 After removal of low molecular weight proteins and other impurities through the diafiltration process, different adsorbents (2%), including the activated carbons and alumina, were used for the removal of endotoxins, which were induced either in the processing broth or from product sources and are known to be a source of pyrogenicity in humans. Different amounts of endotoxin absorbed onto the adsorbents depending on the types of adsorbents. Among the employed absorbents, alumina with the smallest BET value (103) removed the highest level of endotoxins (99.7%) (Table 3). The other activated carbon absorbents such as Norit C Extra USP, Noirt CN1 and Darco KB-B removed significantly less endotoxins, ranging from 88 to 92%. Norit SX Plus and 1G, Norit C Gran and Norit GAC 1240^+^ were not evaluated in regards to removal of residual endotoxin because of the residual colors of Norit SX Plus and 1G, membrane filter blockage by Norit C Gran and metal corrosion by Norit GAC 1240^+^.

**Table 3:** **The percentage of endotoxins and proteins present after the adsorption process**

	Endotoxin (initial: 67.44 EU/*Ml*)		Proteins (initial 77.73 μg/*Ml*)		Remark
Adsorbents (2%)	Residual (EU/*Ml*)	Removal (%)	Residual (μg/*Ml*)	Removal (%)	
Darco KB-B	7.89	88.30	2.82	96.37	-
Norit CN1	6.70	90.07	2.01	97.41	-
Norit C Extra USP	5.40	91.99	2.09	97.32	-
Norit A Supra EUR	6.34	90.60	2.67	96.57	
Norit SX Plus	-	-	-	-	Colors
Norit SX 1G	-	-	-	-	Colors
Norit C Gran	-	-	-	-	No filtration
Norit GAC 1240^+^	-	-	-	-	Metal corrosion
Alumina	0.20	99.70	9.41	87.89%	-

##### Removal of proteins

 High molecular weight proteins, which are metabolic byproducts in this study, were still present in the processing broth. While low molecular weight proteins were removed during the previous diafiltration process using a membrane with a molecular weight cut-off of 50 KDa, high molecular weight proteins needed to be removed using adsorbents. In contrast to endotoxin removal, alumina removed only 87.8% of the proteins, while the activated carbons such as Darco KB-B, Norit CN1, Norit A Supra EUR and Norit C Extra USP removed 96.3 to 97.4% of the proteins. Among the employed absorbents, Norit CN1 removed the most high molecular weight proteins (97.4%) (Table 3), but this increased rate of removal was not significantly different when compared to the other activated carbons such as Darco KB-B, Norit C Extra USP and Norit A Supra EUR. While Darco KB-B and Norit CN1 were acidic, the Norit C Extra USP and Norit A Supra EUR were neutral. In addition, Norit CN1 and Norit C Extra USP had lower methylene blue adsorption (29 and 22 g/100 g, respectively) and Darco KB-B and Norit A Supra EUR had relatively higher values (41 and 42 g/100 g, respectively). We excluded some activated carbon adsorbents from these experiments such as Norit SX Plus and 1G, Norit C Gran and Norit GAC 1240^+^ for the same reasons described in the endotoxin removal experiments, i.e. observation of colors remained.

##### Removal of nucleic acid

 Removal of nucleic acids in the processing broth was also evaluated by employing various adsorbents. The results measured by the UV spectrometer at the wavelength of 260 nm showed that almost all the residual nucleic acids were removed regardless of the state of the activated carbons (powders vs. granule forms). The pH values of the activated carbons did not significantly affect removal of endotoxins, which ranged from 88.3 to 92.0%. The pH values of the Darco KB-B and Norit CN1 were acidic with a ≤ 15 moisture %, and the pH values of Norit C Extra USP and Norit A Supra EUR were neutral with a ≤ moisture 10%. Up to 99.7% of the residual nucleic acids were removed when those activated carbon adsorbents were used. In contrast, alumina removed only 85% of the initial residual nucleic acids. By comparing the removal of both high molecular weight proteins and nucleic acid by the activated carbons and alumina, a higher surface to mass ratio, BET, seemed to result in a higher removal of proteins and nucleic acids. Since the activated carbons had a relatively high BET, i.e. higher surface to mass ratios, ranging 1200 to 2050 m^2^/g, and alumina had a BET value of 103 m^2^/g, alumina seemed to be effective in removal of proteins and nucleic acids.

##### Removal of used adsorbents

**Figure 4:**
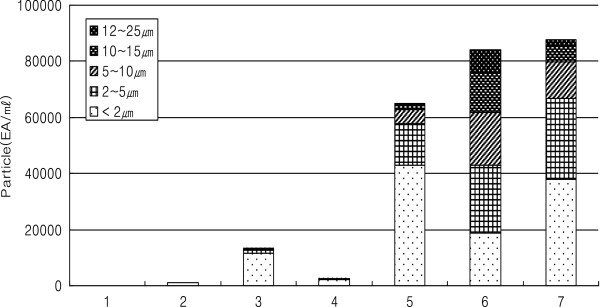
**Particle numbers and sizes under the different conditions described in Table** 4 **.**

 After HA purification, the used adsorbents needed to be removed by filtration to obtain the pure HA products. Even though all the activated carbons were removed from the processed broth, Norit C Gran, which is a granular activated carbon, blocked the filter during filtration when a 1% broth sample was used. To better understand the reason for this result, particles sizes and their distributions were measured for samples containing 2% Norit C Gran and 0.3% hyaluronic acid after stirring for 5 hr (Table 4 and Figure 4). When Norit C Gran without the addition of HA and stirring was used, the broth was easily filtered through a 0.45 μm filter (PR sterile 40, Begerow Inc., Germany) and only 108 microparticles were observed with a diameter of 2 μm (83%). When we added HA (3%) without Norit C Gran adsorbents, the particles seemed to have become entangled, and the numbers of particles with higher sizes increased from 108 to 1,196. When we added the granular activated carbons to the HA solution, the number of particles with higher sizes increased by a factor of two from 1,196 to 2,642. The particle sizes were, however, still mostly less than 5 μm, indicating that the broth obtained was still filterable in this experiment. Significant increases in particle sizes and numbers were observed when we stirred fermented broth containing granular activated carbons for 5 and 13 hrs. 65,151 particles were generated by stirring for 5 hr and their numbers increased to 84,010 after 13 hr of stirring. Furthermore, particles with sizes greater than 5 μm also increased from 12% to 49% under these conditions. Based on these results, Norit C Gran and 5 hr of stirring clearly had an effect on the particle sizes and number and 87,713 particles with smaller sizes were generated and 24% of these were larger than 5 μm in size. These results indicated that under stirring conditions, granular activated carbons broke down. Overall, we did not observe significant effects of pH, BET, methylene blue adsorption on the removal of high molecular weight proteins, nucleic acid and endotixins. However, the types of activated carbons and species of adsorbents significantly affected their removals of those impurities. HA had an effect on their breakdowns and entanglement with HA resulted in an increase in particle size, which lead to blockage of the membrane’s pores.

**Table 4:** **Adsorbent particles and their size distributions, dependent upon the conditions of HA contents and stirring time during purification process by Norit C Gran**

	Norit C Gran. (%)	HA contents (%)	Stirring (hr)	Filtration	Particle(ea/mL, 1/100 dilution)					
					< 2 μm	2 ~ 5 μm	5 ~ 10 μm	10 ~ 15 μm	12 ~ 25 μm	Total
**1**	0	0	0	O	90 (83%)	13 (12%)	4 (4%)	1 (1%)	0	108
**2**	0	0.3	0	O	925 (77%)	178 (15%)	69 (6%)	21 (2%)	3 (0.3%)	1,196
**3**	2	0	0	O	11,513 (87%)	1,301 (10%)	333 (3%)	91 (0.7%)	24 (0.2%)	13,262
**4**	2	0.3	0	O	2,251 (85%)	292 (11%)	67 (3%)	24 (1%)	8 (0.3%)	2,642
**5**	2	0.3	5	X	43,100 (66%)	14,748 (23%)	5,047 (8%)	1,702 (3%)	554 (1%)	65,151
**6**	2	0.3	13	X	18,898 (22%)	23,986 (29%)	18,685 (22%)	14,451 (17%)	7,990 (10%)	84,010
**7**	2	0	5	O	37,932 (43%)	28,703 (33%)	13,234 (15%)	5,576 (6%)	2,268 (3%)	87,713

### Properties of the precipitated & dry HA polymers

White HA powders were obtained using sequential processing of precipitation of HA with 2 times acetone and then drying in clean bench. The final yield of HA was measured to be about 60% when comparing the weight of dry HA with that of the diafiltered dry weight. Its intrinsic viscosity of the HA samples was measured to be 15–17 dL/g with a molecular weight of approximately 1,000 kDa.

### Biocompatibility of HA solution

**Figure 5:**
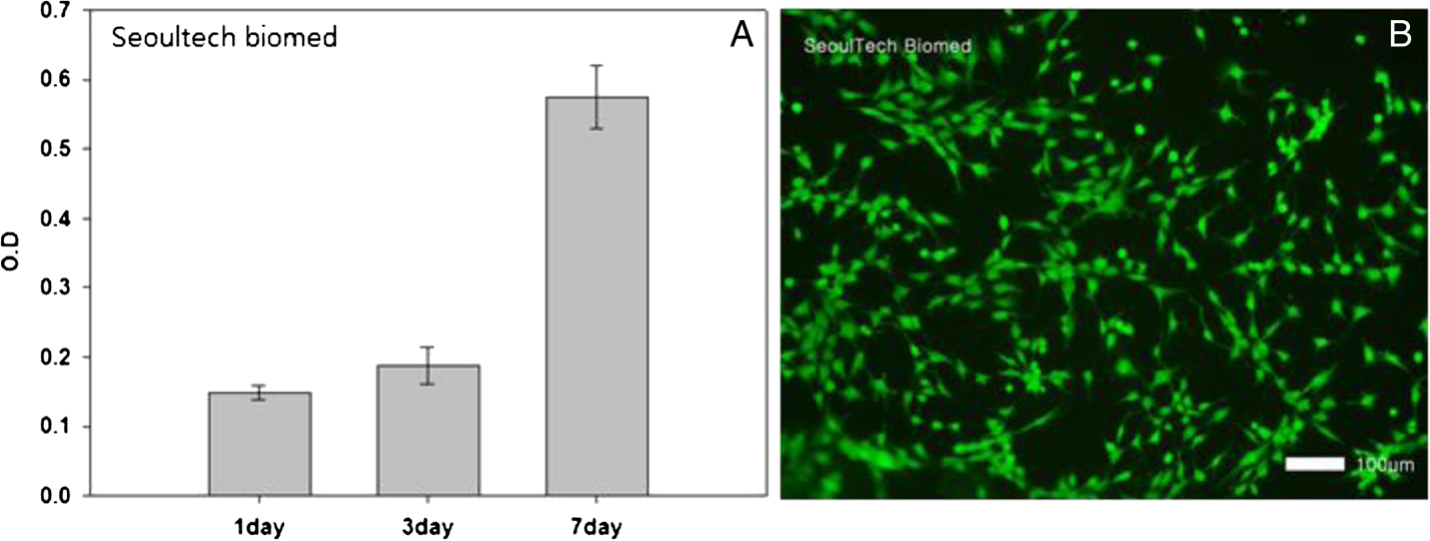
**Proliferation of MC3TC on purified HA solution with assays of CCK-8 over 7 days (A) and live and dead assay (B) after** ***in vitro*** **cell culture for 7 days (B, ×100).**

Biocompatibility of HA solution were excellent in terms of cell proliferation and viability as observed by measurement of CCK-8 and observation of their cell morphologies (Figure 5). The optical density of the samples increased from 0.15 to 0.19 to 0.58 as measured by CCK-8 (Figure 5A) and all the cells were viable without observation of any cell death (Figure 5B).

## Conclusions

We obtained white hyaluronic acid by the processes of fermentation and purification of the fermented broth with filtration and by the use of adsorbents. Removal of the fermented bacterial strains and insoluble impurities was achieved by employing both centrifuge and filtration using filters with a diameter of 0.6-1.5 μm and through the addition of 3 ~ 5% earth soils. Low molecular weight impurities were successfully removed by the ultrafilter with a molecular cut-off of 50 KDa after repeated processes. When we increased the recycling numbers, the permeances of the processing broth increased but the membrane conductivity and lactate concentration decreased, which indicated successful removal of impurities in the broth. Further purification with adsorbents removed nearly all the endotoxins, high molecular weight proteins and nucleic acids from the fermented broth. While alumina was the best adsorbent for the removal of endotoxins, activated carbons such as Norit KBB, CN1, C Extra USP, A Supra EUR were very effective in the removal of high molecular weight proteins and nucleic acids. The granular activated carbons, Norit C Gran, were not adequate for the purification of HA from fermenting broth due to the breakdown of the particles and subsequent entanglement with HA. These two effects result in the formation of a significant amount of large particles, which block the filtering membrane. The other activated carbon in granules, Norit GAC 1240^+^, induced metal corrosions, which is not adequate as an adsorbent. Among the employed activated carbons, the activated carbons in powders such as Norit C Extra USP and Norit A Supra EUR with a higher BET and neutral pH satisfied the criteria required for pharmaceuticals by EU. The results of HA purification using activated carbons indicated that the adsorbent morphology, species and type as well as processing modes such as either with or without stirring were critically important in obtaining high quality HA. The high quality HA could be employed as a biomaterial for pharmaceuticals, tissue engineering and medical devices as well as a carrier for cell therapy. The results of *in vitro* cell culture on the HA solution obtained by mass scale production demonstrated its excellent cell adhesion and proliferation as well as cell viability. These results indicated that the choices of adequate purification processes were important in obtaining biocompatible HA and the purified HA polymers were excellent candidate, respectively, for their applications to biomaterials.

## Availability of supporting data

The data sets supporting the results of this article are included within the article.

## Abbreviations

HA: Hyaluronic acid
BET: Brnauer, Emmett & Teller
CCK-8: Cell counting kit-8
Da: Dalton
MALS: Multi Angle Laser Light Scattering
HPLC: High performance liquid chromatography.
